# Performance measures of racially underrepresented Ph.D. students in biomedical sciences: The UAMS IMSD Program Outcomes

**DOI:** 10.1371/journal.pone.0246683

**Published:** 2021-02-08

**Authors:** Tremaine B. Williams, Latrina Y. Prince, Antiño R. Allen, Kristen M. Sterba, Billy R. Thomas, Robert E. McGehee

**Affiliations:** 1 Graduate School, University of Arkansas for Medical Sciences, Little Rock, Arkansas, United States of America; 2 College of Pharmacy, University of Arkansas for Medical Sciences, Little Rock, Arkansas, United States of America; 3 Department of Institutional Research, University of Arkansas for Medical Sciences, Little Rock, Arkansas, United States of America; 4 Department of Pediatrics, College of Medicine, University of Arkansas for Medical Sciences, Little Rock, Arkansas, United States of America; Indiana University, UNITED STATES

## Abstract

The purpose of this study was to identify performance measures of racially underrepresented minority (RUM) Ph.D. trainees who needed additional training initiatives to assist with completing the UAMS biomedical science degree. A sample of 37 trainees in the 10-year NIH-NIGMS funded Initiative for Maximizing Student Development (IMSD) program at the University of Arkansas for Medical Sciences (UAMS) were examined. Descriptive statistics and correlations examined process measures (GRE scores, GPAs, etc.) and outcome measures (time-to-degree, publications, post-doctoral fellowship, etc.) While differences were found, there were no statistically significant differences between how these two groups (Historically Black Colleges and Universities (HBCUs) and Predominately White Institutions (PWIs)) of students performed over time as Ph.D. students. Graduates who scored lower on the verbal section of the GRE also had a higher final graduate school grade point average in graduates who received their undergraduate training from HBCUs. Of the graduates who received their undergraduate training from PWIs, graduates who scored lower on the quantitative section of the GRE had higher numbers of publications. These findings stimulate the need to 1) reduce reliance on the use of the GRE in admission committee decisions, 2) identify psychometrically valid indicators that tailored to assess outcome variables that are relevant to the careers of biomedical scientists, and 3) ensure the effective use of the tools in making admission decisions.

## Introduction

The National Institute of Health’s (NIH) National Institute of General Medical Sciences (NIGMS) (2015) strategic plan clearly articulated its support for cultivating the next generation of biomedical researchers by centering on creative, highly skilled, and diverse populations. Furthermore, to boost the United States (US) global competitiveness and undertake the large-scale dilemmas health communities grapple with daily requires disparate, prolific, and inventive aptitudes from individuals with contrasting experiences. Through investments in training initiatives, NIGMS currently promotes increasing the number of underrepresented (UR) groups in biomedical research. The National Institute of Health (NIH, 2020), further explicates recognizing that the greatest source of untapped talent is within historically racially UR populations such as African Americans/Blacks, American Indians, and Alaska Natives, as well as Hispanics/LatinoX and Native Hawaiians and Pacific Islanders [1]. Through the nationally recognized need for minority scientists, the potential heterogeneous contributions to biomedical research environments reflect the innovative perspectives of racially UR groups in workforce personnel. For example, teams that are more racially diverse help to develop groups with more persuasive critical thinking skills [2], and often lead to publications in higher-quality science journals [3].

With increased investments in preparatory programs, critical questions regarding effective training mechanisms are currently being explored. These questions range from 1) how to increase diversity in the biomedical research workforce at pipeline entry to 2) vetting the systems that support racially UR trainee’s attainment of a junior faculty position in the biomedical research workforce. The literature consistently references pipeline “leaks” in the development of racially underrepresented scientists [4–8]. The “leaks” in pathways for entering the workforce have generally referred to the barriers encountered or opportunities available to racially UR trainees as they pursue graduate education and career development. According to the National Science Foundation’s Survey of Earned Doctorates, US higher education institutions in 2017 awarded 8,477 biomedical science-related doctoral degrees, but only six percent of trainees were from UR populations [9]. These humbling statistics are an indication of the need for increased attention at the doctoral training stage of career development. More specifically, these results call for urgency in enhancing retention rates and understanding the factors influencing RUM trainees’ matriculation through US doctoral biomedical science degree programs. Therefore, the study’s primary research question seeks to identify performance measures of racially underrepresented minority (RUM) Ph.D. trainees who needed additional training initiatives to assist in completing the UAMS biomedical science degree.

### Historical measures of performance in doctoral training

To better understand factors influencing biomedical science Ph.D. program retention rates, this study focused on potential measures of academic success: undergraduate grade point average (GPA); first-semester graduate GPA; and Graduate Record Examination (GRE) verbal, quantitative, and writing scores. Much controversy has surrounded these factors as accurate measures of long-range productivity in academic and career development (i.e., doctoral degree completion, post-graduation publication productivity, post-graduation extramural grant funding). Kuncel *et al*. found that undergraduate GPA and GRE scores are useful predictors of doctoral grade point average, first-semester graduate GPA, and publication productivity (N = 1,753) [10]. However, Petersen *et al*. found that GRE scores failed to predict students’ time-to-degree completion and who would abandon their STEM doctoral programs in the first year (N = 1,805) [11]. Sealy *et al*. found evidence that there is no relationship between GRE scores and time-to-degree completion, predoctoral fellowship awards, and post-graduation outcomes such as first-authored publications (N = 28) [12]. Moneta-Koehler *et al*. found GRE scores did not predict graduation rates, qualifying exam success, time-to-final dissertation defense, conference presentation acceptance, first-authored publications, or success in securing grants (N = 683) [13].

Despite the evidence presented by the previously mentioned studies, the Educational Testing Service (ETS) which administers the GRE suggests that GRE scores are the only measure that provides a standardized and objective approach to comparing students from different backgrounds [14]. However, Miller and Stassun (2014) questioned the validity of the GRE’s ability to consider the ethnic, racial, and socioeconomic experiences of UR groups [15]. In addition, others found that GRE scores are not accurate measures of graduate school success [13].

Another viable predictor of doctoral training success that is often used by program administration is first semester Ph.D. GPA. Researchers have argued that first-year grades do not represent the bulk of students’ performance in subsequent years during graduate preparation [16, 17]. Yet, the reliance on GPA during the first-semester and the first year has persisted as measures of success [13, 18]. With the conflicting levels of value placed on aptitude scores and other performance measures, biomedical sciences doctoral-level training programs could benefit from conducting more thorough, individual examinations of performance measures related to the success of students from racially underrepresented groups; further motivating the need for this study.

### The UAMS Initiative for Maximizing Student Development (IMSD)

The University of Arkansas for Medical Sciences (UAMS) is the state’s only comprehensive academic health center. Each year, more than 2,800 trainees enroll at UAMS and 800 resident physicians complete their training at UAMS or one of its eight Regional Programs around the state. UAMS, which also includes the hospital and all outpatient clinics, treats patients throughout Arkansas, the United States, and the world. UAMS is Arkansas’ largest basic and applied research institution, with internationally renowned programs in multiple myeloma, aging, cancer, psychiatry, ophthalmology, and informatics. UAMS averages more than $170 million in annual research funding, grants, and contracts and is a member of the NIH National Center for Advancing Translational Sciences (NCATS) Clinical and Translational Science Awards (CTSA) consortium that supports the university’s Translational Research Institute (TRI) and the Arkansas IDeA Network of Biomedical Research Excellence (Arkansas INBRE). UAMS is classified as a public “Special Focus Institution-Medical Schools and Centers” by the Carnegie Classification System and includes the Graduate School and five professional schools: Colleges of Medicine, Nursing, Pharmacy, Health Professions, and Public Health. In the past five years, an average of 8% of tenure-track faculty have been from racially underrepresented (UR) groups and total enrollment by UR trainees in biomedical research doctoral programs averaged 21% of the student population during the 10-year implementation of the UAMS IMSD program (2008–2018).

Funded by the National Institutes of Health (NIH), National Institute for General Medical Sciences, the specific aims of the IMSD program is to increase the diverse pool of individuals earning a biomedical science Ph.D. and to assist these individuals in transitioning into research workforce careers. Through the program, first-generation students, students with disabilities, and individuals from racially underrepresented groups (i.e. Blacks/African Americans, Hispanics/LatinX, American Indians/Alaska Natives, and Native Hawaiians and other Pacific Islanders) have access to more resources that assist in being successful in graduate school. The program offers a set of curricular and co-curricular initiatives. These activities are 1) a formal peer mentoring: 2) a five-week summer transition program; 3) competency-based academic portfolio management; 4) a seminar series highlighting minority role models, and 5) active learning-based biochemistry course. The overall goals of the UAMS IMSD program are to 1) enhance success in retaining UR trainees and 2) refine strategies to recruit additional UR trainees through a set of predefined initiatives. More specifically, the objective of the program was to increase UR doctoral trainee enrollment as a percent of all doctoral enrollment to 20% by 2019 and to graduate 90% of the admitted trainees. Currently, UR trainees make up 17% of doctoral trainees enrolled in UAMS biomedical research programs with a mean GPA of 3.25 over the 10 year period of administering the initiatives through the UAMS IMSD program. These initiatives aligned with the NIH Minority Biomedical Research Support (MBRS) program goals which are devoted to increasing the number of UR trainees who graduate from doctoral programs in the biomedical sciences at institutions with a research-intensive environment; further reducing the Ph.D. completion gap between UR and non-UR trainees in the biomedical sciences.

### UAMS IMSD interventions

#### The Ph.D. summer transition program

The Ph.D. Summer Transition Program is required for all trainees admitted to the UAMS IMSD program in the Summer prior to their first semester of graduate study. The Summer intervention consists of a 5-week intensive review covering basic concepts in biochemistry, time management, communication, and other academic skills. Throughout the five weeks, trainees are actively engaged with previously accepted UAMS IMSD trainees and biomedical research faculty. Each week’s curriculum demands approximately 15 hours of formal lectures in fundamental biomedical research areas. Compulsory courses include molecular biology, systems biology, and data management; DNA sequences, replication, genomics, and comparative sequencing; RNA sequences, transcription, transcriptomics, rRNA and phylogeny; protein sequences, proteomics, and data management; and enzymes, metabolism, biological functional classification categories. Students also devote twenty hours per week to rotating through at least one of the biomedical research labs categorized by the subdomains of biomedical sciences: biochemistry and molecular biology; cell biology and physiology; microbiology and immunology; neuroscience; pathobiology; and pharmacology, toxicology, and experimental therapeutics. The remaining 10 hours are dedicated to attending psychosocial seminars on cohort and community building, campus resource and culture acclimation, and learning strategies: aiming to develop a holistic set of additional competencies beyond sciences.

#### Active learning-based biochemistry course

All IMSD trainees are required to take a graduate-level course in biochemistry. The course is intentionally designed to actively engage students in the learning experience. An active learning component consisting of micro-level chapter goals is used to aid students’ cognitive processing of the concepts and to address the needs of students who learn sequentially. Strategies also include problem-based, small group class discussions. The design of these learning exercises concentrates on developing students’ delivery of material, insights, and reasoning related to the lectures, assignments, and group discussions. The instructor provides feedback and resources to correct students’ misinterpretations of biochemistry concepts.

#### Competency-based academic portfolios

The IMSD portfolio program supports UAMS’ mission to teach, to heal, to search, and to serve. In addition, the portfolio program supports the IMSD mission to increase the number of UR trainees graduating from biomedical doctoral programs, thereby reducing the Ph.D. completion gap between UR and non-UR trainees. The portfolio program contributes to reducing the Ph.D. completion gap by teaching trainees how to explicate the attainment of skills. To facilitate career development, each IMSD trainee creates a competency-based academic portfolio used for both formative and summative assessment. As a formative assessment tool, the portfolio is used to document increases in skill acquisition. As a summative assessment, the portfolio is a demonstrates competency in specific areas such as biochemistry and informatics. These tools teach management processes for skill acquisition: expanding competencies to be more holistic and reflective of those needed to support long-term career goals.

#### Peer mentoring program

The UAMS mentoring program plays a vital role in supporting UR student development of knowledge and competencies (both science and non-science) needed for success in academia. Mentoring significantly influences the successful research and career trajectories of Ph.D. students in securing post-doctoral fellowships; critical to securing a basic faculty position. The Peer Mentoring Program (PMP) provides an experienced upper-level graduate student as a resource for guiding and orienting new trainees to the culture of biomedical science research. The PMP offers interpersonal and intrapersonal support to nurture the mentee’s professional development. The Peer Mentor is an upper-level student who has completed their course work and candidacy exams. They serve in a collegial and supportive role by sharing past experiences and offering directions based on first-hand Graduate School experiences. The PMP also supports trainee needs for guidance related to career goals, resources, and content/interaction skills.

## Methods

The University of Arkansas for Medical Sciences’ Institutional Review Board reviewed and approved this study (Protocol# 260465). Below are several hypotheses that were formulated based on findings in the literature [11, 12, 15] and needs for program evaluation. These hypotheses were designed to support the central research question involving identifying performance measures of racially underrepresented minority (RUM) Ph.D. trainees who needed additional training initiatives to assist with completing the UAMS biomedical science degree.

■ There will be no relationship between UR trainees’ first-semester Ph.D. GPA and GRE scores.
■ There will be no relationship between UR trainees’ first-semester Ph.D. GPA and undergraduate GPA.
■ There will be no differences in performance between UR trainees with undergraduate training at HBCUs and UR trainees with undergraduate training at PWIs.

Quantitative, verbal, and analytical writing scores on the GRE, along with undergraduate GPA and first-semester graduate school GPA were collected on 37 UAMS IMSD program trainees. These variables were selected because they are generally used by biomedical science faculty to determine if a UR applicant should be admitted or matriculate through a Ph.D. program in biomedical sciences. For example, an applicant who earns a 1.0 (out of 4.0) first-semester Ph.D. GPA may not continue to the second semester because of potential institutional policies related to increasing GPA within a certain timeframe. Additionally, Ph.D. student performance was also examined in the context of prior training at HBCUs versus PWIs. Descriptive and correlative statistics were used to examine potential predictor variables such as undergraduate GPA, graduate GPA, and GRE Scores. For one variable to predict another, the variables must have some level of association as reflected by a linear relationship [19]. Pearson’s *r* was used to determine the extent to which process variables were associated (i.e., GRE scores, undergraduate GPA, etc.) in the sample of 37 students and graduates. Additionally, Spearman’s Rho was used to determine the extent to which process variables (i.e., GRE scores, undergraduate GPA, etc.) with the outcome variables (GPA, time-to-degree, publications, etc.) in the sample of 18 graduates. The Mann-Whitney U test was used to compare scores by demographic variable (HBCUs vs. PWIs) to assess significant differences between groups. Four statistical assumptions of the Mann-Whitney U test exist [20]. All assumptions, including the lack of distribution normality, were met for all variables.

## Results

Since the program began in 2009, forty-eight students have been accepted. The population of accepted trainees includes current graduate school students (n = 24), Ph.D. graduates (n = 18), Master of Science degree graduates (n = 3), and academic withdrawals (n = 3). Of the 48 trainees, complete admission packet data for 37 trainees were available at the time of this study. Table 1 presents the demographic information for the 37 trainees whose ages ranged between 21 and 26.

**Table 1:** Racial identity of 37 students and graduates.

RACE	MALE	FEMALE
**AFRICAN AMERICAN/BLACK**	7	22
**CAUCASIAN/WHITE**	1	2
**HISPANIC/LATINX**	2	1
**ASIAN AMERICAN**	1	0
**TOTAL**	11	26

Descriptive statistics on quantitative, verbal, and analytical writing scores on the GRE, along with undergraduate GPA and first-semester graduate school GPA are presented on 37 IMSD program trainees *(*Table 2*)*.

**Table 2:** Descriptive statistics for 37 IMSD students and graduates.

VARIABLES	RANGE	MIN.	MAX.	MEAN	STD. DEV.
**FIRST SEM. GRS GPA**	2	2	4	3.25	0.48
**GRE-V**	30	130	160	148	6.38
**GRE-Q**	23	136	159	146	4.95
**GRE-W**	3	2	5	3.5	0.72
**UGRD GPA**	1.38	2.62	4	3.29	0.31

**Fig 1:**
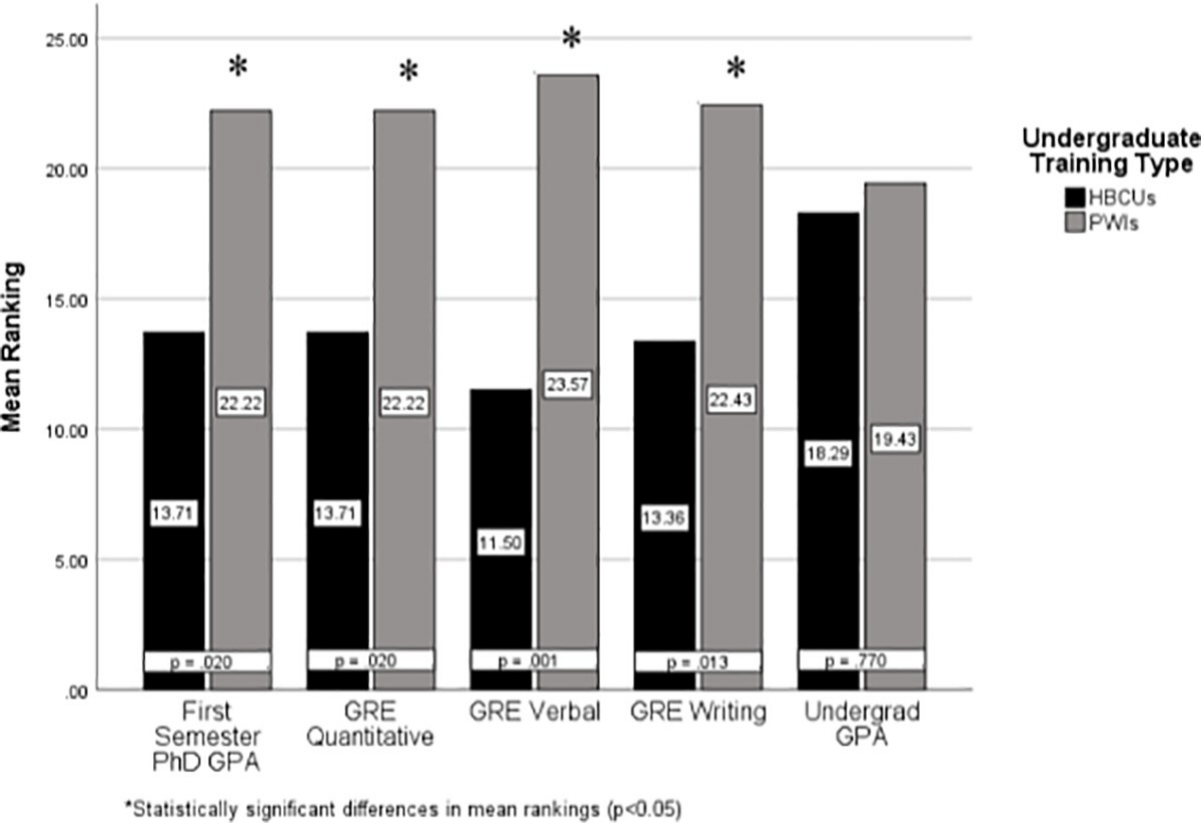
Mann-Whitney U mean rankings of UR trainees: PWIs (N = 23) and HBCUs (N = 14). Given the lack of normality in the distributions of GRE Scores and undergraduate GPA, a Mann-Whitney U test was used to examine differences between the two groups. Fig 1 presents the mean rakings across program admission variables for the 37 IMSD students. Mean rankings of the Mann-Whitney U test were interpreted because histograms confirmed differences in the distribution shape of the trainees from HBCUs and trainees from PWIs [20]. Therefore, there are differences in first-semester Ph.D. GPA between UR trainees with undergraduate training at HBCUs and UR trainees with undergraduate training at PWIs.

*GRE-V = GRE Verbal Score; GRE-Q = GRE Quantitative Score; GRE-W = GRE Writing; UGRD GPA = Final Cumulative Undergraduate Grade Point Average; FIRST SEM. GRS GPA = First Semester Graduate School Grade Point Average;

According to the mean ranking presented in Fig 1 above through the Mann-Whitney U test, UR trainees who were trained at PWIs had higher grade point averages during their first semester in the Ph.D. program than UR trainees from HBCUs. This difference is represented by the 22.22 and 13.71 mean rankings in Fig 1. The *p-value* of 0.02 reflected in Fig 1 is below the α-level of .05, which indicated the results were significant. Therefore, differences in first-semester Ph.D. GPA exists between UR trainees with undergraduate training at HBCUs and UR trainees with undergraduate training at PWIs. Additionally, there were differences in GRE verbal, quantitative, and writing scores between UR trainees with undergraduate training at HBCUs and UR trainees with undergraduate training at PWIs. Given the *p-values* resulting from the Mann-Whitney U test presented in Fig 1, differences in GRE verbal, quantitative, and writing scores between UR trainees with undergraduate training at HBCUs and UR trainees with undergraduate training at PWIs were also statistically significant. However, the mean ranking for undergraduate GPA between UR trainees with undergraduate training at HBCUs and UR trainees with undergraduate training at PWIs was 18.29 and 19.43, respectively. With an α level of 0.77, as presented in Fig 1, differences in UR trainees with undergraduate training at HBCUs and UR trainees with undergraduate training at PWIs were not statistically significant.

Descriptive statistics on program admission and matriculation variables are presented for the 18 graduates of the IMSD program (Table 3*)*.

**Table 3:** Descriptive statistics of 18 IMSD graduates.

VARIABLES	RANGE	MIN.	MAX.	MEAN	STD. DEV.
**GRE-V**	22	138	160	147.78	5.71
**GRE-Q**	15	137	152	145.89	4.16
**GRE-W**	2.5	2.5	5.0	3.47	.63
**UGRD GPA**	1.17	2.62	3.79	3.25	.29
**FIRST SEM. GRS GPA**	1.50	2.50	4.00	3.28	.43
**FINAL GRS GPA**	.77	3.23	4.00	3.60	.23
**TIME -TO-DEG**	36	40	76	55.78	10.82
**PUBS**	17	0	17	5.39	4.88
**FIRST-AUTHED PUBS**	5	0	5	1.61	1.42
**POSTDOC**	1	0	1	.67	.49

*GRE-V = GRE Verbal Score; GRE-Q = GRE Quantitative Score; GRE-W = GRE Writing; UGRD GPA = Final Cumulative Undergraduate Grade Point Average; FIRST SEM. GRS GPA = First Semester Graduate School Grade Point Average; FINAL GRS GPA = Final Graduate School Grade Point Average; TIME-TO-DEG = Time-to-degree in Months; PUBS = Number of PubMed Publications by Student/Graduate during Graduate School; FIRST-AUTHED PUBS = Number of First-authored PubMed Publications by Student/Graduate during Graduate School; POSTDOC = Post-Doctoral Fellowships Received

Table 4 shows a Spearman’s Rho correlation coefficient of -0.901 (Sig. = .006; p < .01) between FINAL GRS GPA and GRE-V score. This represents a significant, very strong negative correlation. This means that graduates who scored lower on the verbal section of the GRE also had a higher final grade point average than graduates who received their undergraduate training from HBCUs. Table 4 also shows a Spearman’s Rho correlation coefficient of 0.922 (Sig. = .003; p < .01) between PUBS and FIRST AUTH. PUBS. This represents a significant, very strong positive correlation. This means that the more publications a graduate had (any author level), the more first-authored publications they had (of graduates who received their undergraduate training from HBCUs). Additionally, PUBS and POST-DOC showed a Spearman’s Rho correlation coefficient of 0.828 (Sig. = .02; p < .05): representing a significant, very strong positive correlation. This means that the more publications a graduate had (any author level), the more likely they were to have received a post-doctoral fellowship POST-DOC and GRE-Q had a Spearman’s Rho correlation coefficient of 0.798 (Sig. = .03; p < .05); representing a strong positive correlation. This means that students who scored higher on the quantitative section of the GRE were more likely to have received a post-doctoral fellowship.

**Table 4:** Spearman’s rank correlation of IMSD graduates from HBCUs.

	GRE-V	GRE-Q	GRE-W	UGRD GPA	FIRST SEM. GRS GPA	FINAL GRS GPA	TIME-TO-DEG	PUBS	FIRST AUTH. PUBS	POSTDOC
**GRE-V**	1.000	-0.236	0.500	-0.282	-0.066	-.901**	0.536	-0.425	-0.595	-0.239
**GRE-Q**	-0.236	1.000	0.389	0.273	0.623	0.577	0.036	0.661	0.566	.798*
**GRE-W**	0.500	0.389	1.000	0.296	0.404	-0.330	0.120	-0.019	-0.077	0.163
**UGRD GPA**	-0.282	0.273	0.296	1.000	-0.142	0.342	0.200	-0.349	-0.255	-0.239
**FIRST SEM. GRS GPA**	-0.066	0.623	0.404	-0.142	1.000	0.281	-0.538	0.725	0.490	0.497
**FINAL GRS GPA**	-.901**	0.577	-0.330	0.342	0.281	1.000	-0.360	0.543	0.617	0.474
**TIME-TO-DEG**	0.536	0.036	0.120	0.200	-0.538	-0.360	1.000	-0.519	-0.538	-0.080
**PUBS**	-0.425	0.661	-0.019	-0.349	0.725	0.543	-0.519	1.000	.922**	.828*
**FIRSTAUTH. PUBS**	-0.595	0.566	-0.077	-0.255	0.490	0.617	-0.538	.922**	1.000	.828*
**POST-DOC**	-0.239	.798*	0.163	-0.239	0.497	0.474	-0.080	.828*	.828*	1.000

*Correlation significant at the 0.05 level

**correlation significant at the 0.01 level.

Table 5 shows a Spearman’s Rho correlation coefficient of -0.663 (Sig. = .03; p < .05) between GRE-Q and FIRST AUTH. PUBS. This represents a significant, strong negative correlation. This means graduates who scored lower on the quantitative section of the GRE also had a higher numbers of publications in graduates who received their undergraduate training from PWIs. Table 5 also shows a Spearman’s Rho correlation coefficient of 0.696 (Sig. = .01; p < .05) between FIRST AUTH. PUBS and FIRST SEM. GRS GPA: representing a significant, strong positive correlation. This means graduates who had higher first semester Ph.D. grade point averages also had a higher number of first-authored publications. Additionally, a Spearman’s Rho correlation coefficient of 0.762 (Sig. = .006; p < .01) between PUBS and FIRST AUTH. PUBS. This represents a significant, strong positive correlation. This means that the more publications a graduate had (any author level), the more first-authored publications they had in graduates who received their undergraduate training from PWIs.

**Table 5:** Spearman’s rank correlation of IMSD graduates from PWIs.

	GRE-V	GRE-Q	GRE-W	UGRD GPA	FIRST SEM. GRS GPA	FINAL GRS GPA	TIME-TO-DEG	PUBS	FIRST AUTH. PUBS	POSTDOC
**GRE-V**	1.000	0.233	0.058	-0.082	-0.109	-0.314	-0.234	-0.274	0.019	-0.239
**GRE-Q**	0.233	1.000	-0.508	0.009	-0.502	-0.424	-0.376	-0.537	-.663*	0.092
**GRE-W**	0.058	-0.508	1.000	-0.419	0.088	-0.005	0.157	0.122	0.055	-0.064
**UGRD GPA**	-0.082	0.009	-0.419	1.000	0.588	0.176	0.346	-0.195	0.202	0.389
**FIRST SEM. GRS GPA**	-0.109	-0.502	0.088	0.588	1.000	0.601	0.239	0.406	.696*	0.478
**FINAL GRS GPA**	-0.314	-0.424	-0.005	0.176	0.601	1.000	-0.115	0.574	0.584	0.599
**TIME-TO-DEG**	-0.234	-0.376	0.157	0.346	0.239	-0.115	1.000	0.261	0.134	0.000
**PUBS**	-0.274	-0.537	0.122	-0.195	0.406	0.574	0.261	1.000	.762**	0.330
**FIRSTAUTH. PUBS**	0.019	-.663*	0.055	0.202	.696*	0.584	0.134	.762**	1.000	0.336
**POST-DOC**	-0.239	0.092	-0.064	0.389	0.478	0.599	0.000	0.330	0.336	1.000

*Correlation significant at the 0.05 level

**correlation significant at the 0.01 level.

## Discussion

The purpose of this study was to identify performance measures of racially underrepresented minority (RUM) Ph.D. trainees who needed additional training initiatives to assist with completing the UAMS biomedical science degree. While not all graduate institutions have IMSD programs, this study offers significant benefits to educational institutions with supporting RUM trainees. The scientific contributions to the literature include 1) sharing a framework for evaluating the effectiveness of program-level performance measures, 2) assessing the equity of admission decision practices of individual, biomedical sciences programs and 3) providing support for the need for increasing curriculum alignment initiatives between undergraduate and graduate programs: stimulating the training of scientists from racially underrepresented groups.

Considering that many Ph.D. students struggle academically during the first year of a Ph.D. program, debates have ensued throughout the literature over the use of GPAs collected during the first year as an adequate measure of graduate school degree attainment, time-to-degree, or publication productivity and other outcomes [11, 21–23]. However, the rationale for using undergraduate GPA as a measure is simply due to the significant value that training programs place on GPA to support admission decisions and as measures of whether to allow trainees to progress toward degree attainment. A low GPA during the first-semester could result in dismissal from the Ph.D. program: reinforcing its usefulness as a meaningful measure of academic performance. The removal of Ph.D. programs’ GRE requirements indicates a move toward expanded holistic approaches to evaluating UR Ph.D. applicants and inversely increasing dependence on measures such as GPA.

**Fig 2:**
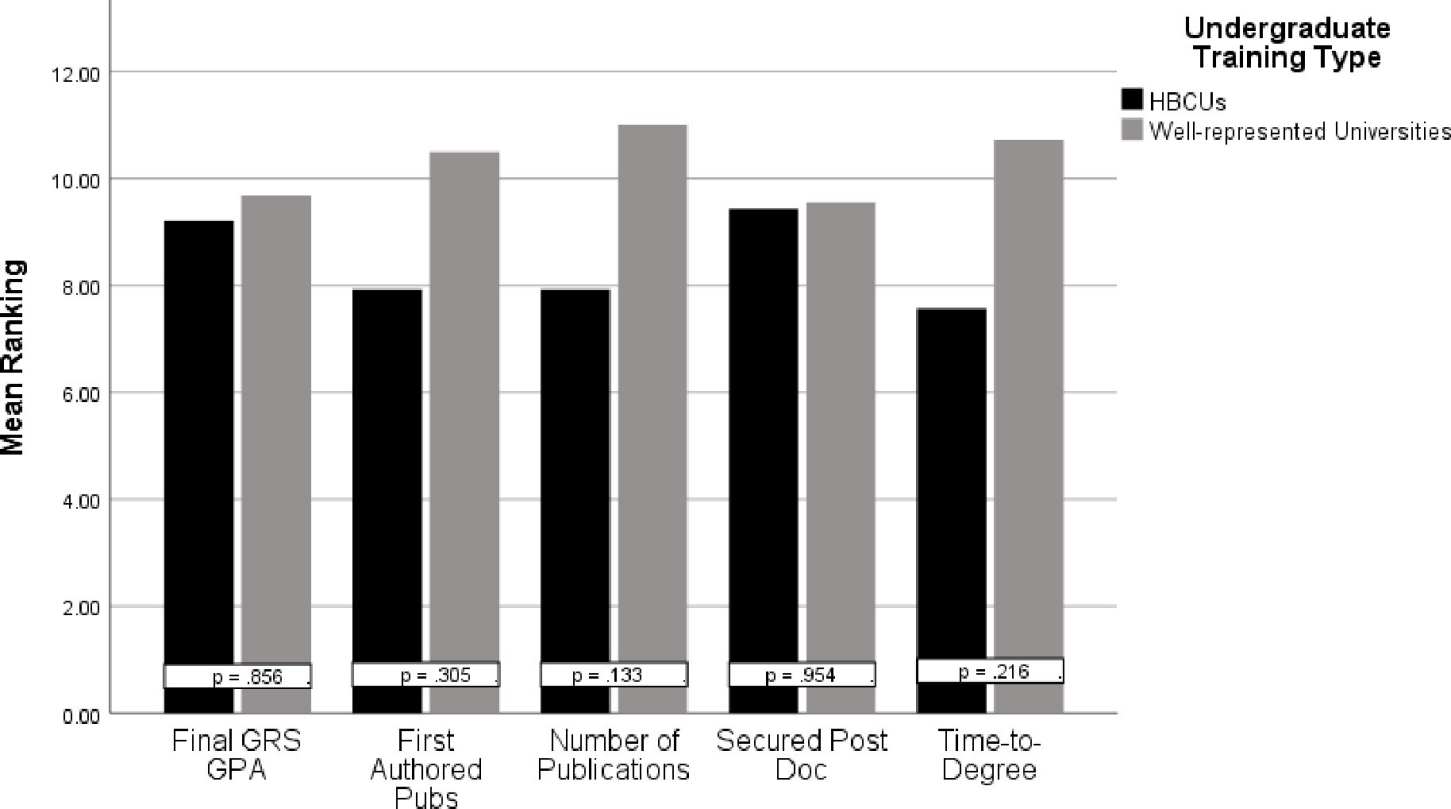
Mann-Whitney U graduate rankings of UR trainees: PWIs (N = 11) and HBCUs (N = 7). A Mann-Whitney U test was also used to examine differences between the two groups in additional matriculation measures in the 18 graduates of the program. Fig 2 presents the mean rakings across these variables. There were no statistically significant differences in matriculation variables between UR graduates with undergraduate training from HBCUs and UR trainees with undergraduate training from PWIs.

A significant finding was the need to acknowledge differences in first-semester Ph.D. GPA and GRE scores between UR trainees with undergraduate training at HBCUs and UR trainees with undergraduate training at PWIs. The analyses found these differences to be statistically significant and meaningful. Therefore, these findings indicate that there is a need to provide additional training initiatives to assist UR trainees who obtain undergraduate degrees from HBCUs to increase first semester Ph.D. GPA. While differences were found, there were no statistically significant differences between how these two groups of students performed over time as Ph.D. students (Fig 2). This indicated that the impacts of undergraduate training could be mitigated and/or supplemented during graduate training; regardless of undergraduate institution type. While the literature is substantially lacking in understanding the influences of undergraduate training on graduate performance in biomedical science Ph.D. trainees, similar findings have been presented in medical students. For example, Capers and Way found African American/Black students (N = 173) at The Ohio State University’s College of Medicine who graduated from HBCUs had lower average MCAT scores than their counterparts from PWIs [23].

Undergraduate curricula (i.e. biology, chemistry) generally seek to provide the fundamentals of biomedical sciences. Each curriculum is unique and dependent on factors such as faculty expertise and resources. Differences in undergraduate curricula presented difficultly in identifying specific variables, including courses that may account for the differences observed in this study. Undergraduate curriculum differences also presented a need for future inquiry to assess STEM curricula at all types of undergraduate institutions to assess the extent to which potential trainees have developed competency in the fundamentals areas that support entry into biomedical science. Differences in undergraduate institution types were in no way a criticism of the HBCU’s in which the students received undergraduate training. It is an acknowledgment of the significant work performed by HBCU faculty in developing trainees for careers in biomedical sciences. HBCUs produce approximately 25% of all Black/African American baccalaureates; 20% of Black/African American baccalaureates degrees in science and engineering [24]. Moreover, approximately 25% of African American STEM Ph.D. recipients received their undergraduate training from an HBCU [25]. A historical component of the graduate school application process is questioning students’ undergraduate training and aptitude, as reflected by undergraduate transcript submission requirements [11–13]. Graduate faculty at PWIs generally use this information to assess the undergraduate training of their graduate students as a measure of determining graduate training preparedness. However, isolated occurrences where one or two students lack knowledge, skills, or abilities in an area fundamental to biomedical science cannot be generalized to reflect the strength and reputation of the undergraduate institution the applicant attended.

Graduate faculty must consider that undergraduate HBCU and other underrepresented serving institutions confront similar challenges when transitioning students from high school to undergraduate training. Many HBCUs have a legacy of serving UR student populations that enter college underprepared [23, 26]: many from urban and rural public school systems in distress. These students often require additional time-to-degree attainment during undergraduate and graduate training. This indicates that there is a need for increased communication and collaboration between graduate and undergraduate programs regarding the teaching of relevant content and the capacity of what can be taught at the undergraduate level, particularly between PWIs and HBCU undergraduate institutions. This level of collaboration could help increase UR trainee preparedness for graduate training and improve UR retention and graduation rates: a primary goal of most graduate institutions.

**Fig 3:**
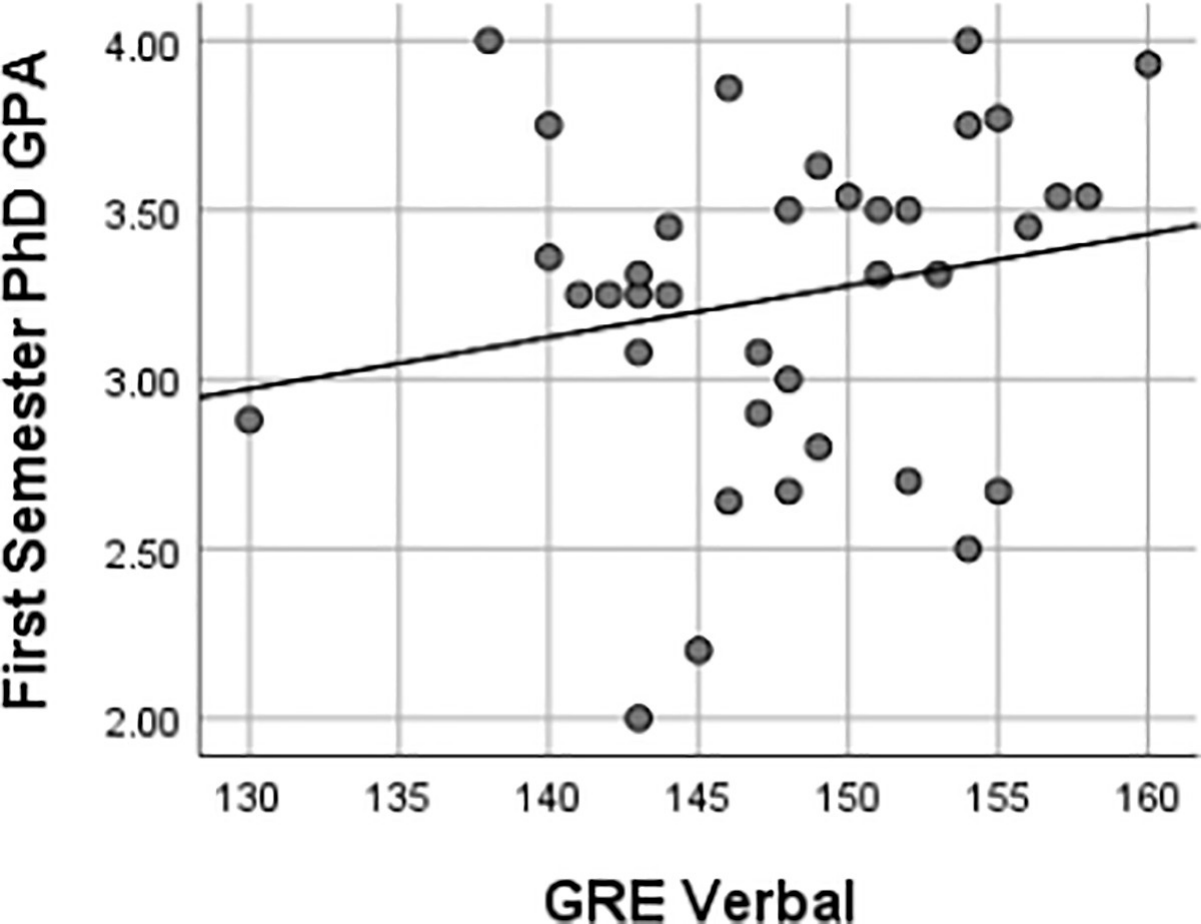
Relationships between first semester Ph.D. GPA & GRE verbal score. Pearson’s correlation coefficient of 0.20 reflected a weak relationship between first semester Ph.D. GPA and GRE verbal score. Given the *p-value* of 0.24 was above the .05 α-level, no significant relationship existed between a UR trainee’s first-semester Ph.D. GPA and their GRE verbal score.

**Fig 4:**
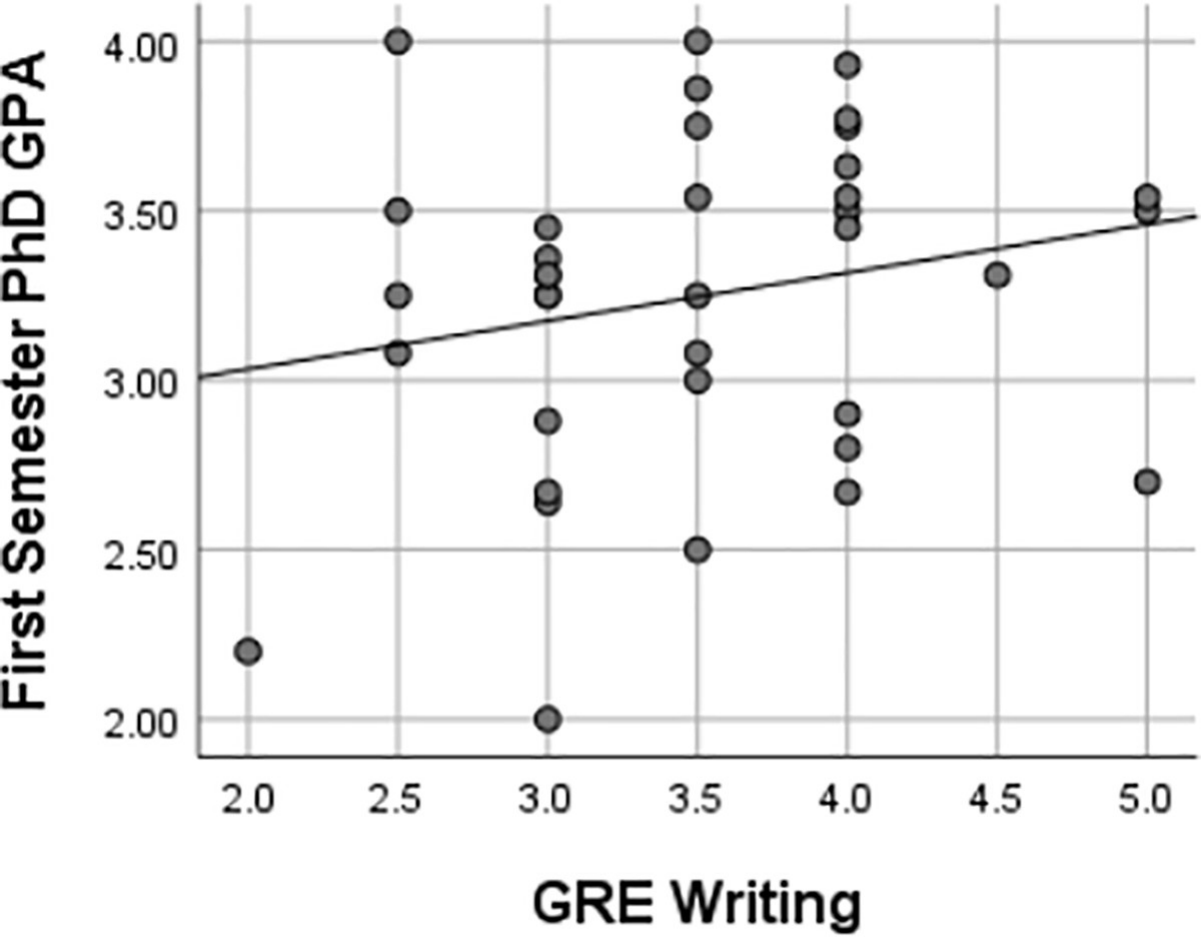
Relationships between first semester Ph.D. GPA & GRE writing score. Pearson’s correlation coefficient of 0.21 reflected a weak relationship between first semester Ph.D. GPA and GRE writing score. Given the *p-value* of 0.21 was above the .05 α-level, no significant relationship existed between a UR trainee’s first-semester Ph.D. GPA and their GRE analytical writing score.

**Fig 5:**
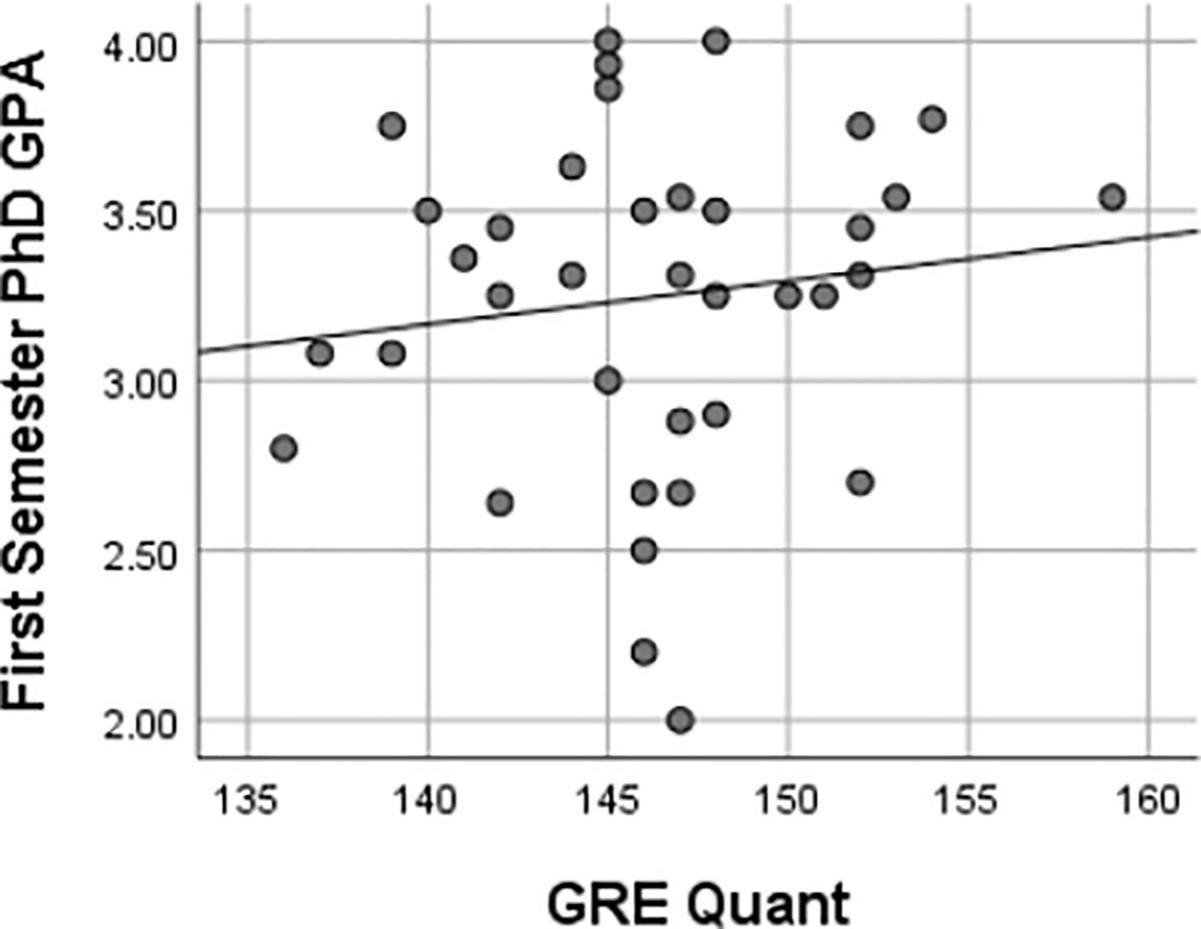
Relationships between first semester Ph.D. GPA & GRE quantitative score. Pearson’s correlation coefficient of 0.13 reflected a weak relationship between the first semester Ph.D. GPA and GRE quantitative score. Given the *p-value* of 0.44 was above the .05 α-level, no significant relationship existed between a UR trainee’s first-semester Ph.D. GPA and their GRE quantitative score.

**Fig 6:**
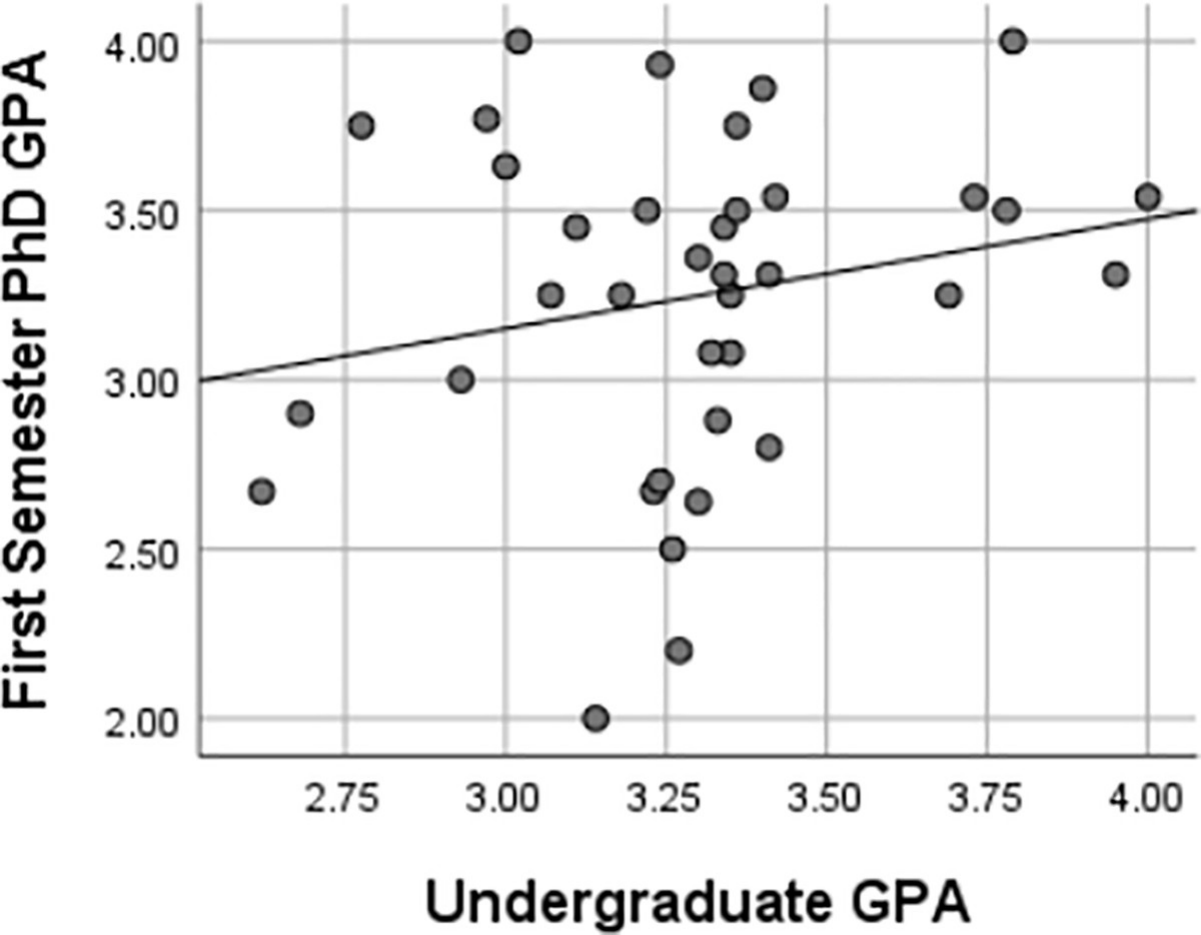
Relationships between first semester Ph.D. GPA & undergraduate GPA. Pearson’s correlation coefficient of 0.21 reflected a weak relationship between first-semester Ph.D. GPA and GRE quantitative score. Given the *p-value* of 0.22 was above the .05 α-level, no significant relationship exists between a UR trainee’s first-semester Ph.D. GPA and their GRE quantitative score.

This study also found that there were weak, insignificant associations between GRE scores and first-semester Ph.D. GPA, as represented in Figs 3–6 using Pearson’s *r*. Furthermore, there were implications regarding the potential ability of GRE scores to predict the first-semester Ph.D. GPA of UR trainees given the lack of association between the variables in this study. Regressions are frequently used to predict performance [27]. The variables must have some level of association as, reflected by a linear relationship, for one variable to predict another [20]. Pearson’s *r* was used to determine the extent to which the response variables (i.e., GRE scores, undergraduate GPA) were associated with performance as reflected by the first semester Ph.D. GPA. If linear regressions were used to predict first-semester GPA based on GRE scores, there would need to be stronger correlations and smaller significance levels than the test statistics produced by the methods in this study. It is improbable that GRE scores could also serve as predictors of UR trainees’ first-semester Ph.D. GPA based on the analyses of this study.

GRE score analysis varied between the two undergraduate training groups (PWI Vs. HBCU) Graduates who scored lower on the verbal section of the GRE also had a higher final graduate school grade point average in graduates who received their undergraduate training from HBCUs (Table 4). Of graduates who received their undergraduate training from HBCUs, we found that graduates who scored higher on the quantitative section of the GRE were more likely to have received a post-doctoral fellowship (Table 4). Of the graduates who received their undergraduate training from PWIs, graduates who scored lower on the quantitative section of the GRE had higher numbers of publications (Table 5). Additionally, graduates who had higher first semester Ph.D. grade point averages also had higher numbers of first-authored publications. These findings substantiate the value of first semester Ph.D. GPA as a strong indicator of performance of graduates who received their undergraduate training from PWIs. Additionally, the more publications a graduate had (any author level), the more likely they were to have received a post-doctoral fellowship. This indicated that the number of publications received during graduate school could be a strong performance indicator for doctoral programs with outcome measures of supporting students in securing post-doctoral fellowships.

The majority of these findings build on the existing body of knowledge [11–13] by supporting previous findings that GRE scores are not strong measures of successful outcomes (i.e. first-semester graduate GPA, final doctoral GPA, qualifying exam success, time-to-degree, graduation rates, publication productivity, conference presentation acceptance, first-authored publications, or success in securing grants). These findings are critical because admission committees generally seek to accept candidates whom have higher quantitative GRE scores; not lower. Therefore, this finding increases the need to 1) reduce reliance on the use of the GRE in admission committee decisions, 2) identify psychometrically valid indicators that are tailored to assess outcome variables that are relevant to the careers of biomedical scientists, and 3) ensure the effective use of the tools in making admission decisions. These recommendations could strengthen the validity of how racially underrepresented minorities could be evaluated for entry and matriculation through doctoral training programs in biomedical sciences.

## Limitations of the study

First, the sample of 37 participants was small for a 10-year period. Yet, the number is higher than other IMSD initiatives related literature: 28 students [12] and 17 students [28]. It is also reflective of the pace at which racially underrepresented, biomedical scientists are trained throughout the United States. Second, the results presented in this study are from a single IMSD initiative; thus, may more reflect the UAMS graduate school’s recruitment approach or IMSD initiatives dynamics. Third, all 37 UR trainees sampled in this study were exposed to the all initiatives of the UAMS IMSD program. The results serve as motivation for additional areas of inquiry that are needed to understand the undergraduate curriculum impacts on a UR trainees’ transition from undergraduate to graduate training. While the results of this study contribute to understanding the performance measures of UR trainees, the results are not generalizable. The findings of this study could be validated through 1) larger-scale studies with multiple IMSD programs and 2) collectively, through assessments of individual IMSD programs.
